# Knockout of the *dhfr-ts* Gene in *Trypanosoma cruzi* Generates Attenuated Parasites Able to Confer Protection against a Virulent Challenge

**DOI:** 10.1371/journal.pntd.0001418

**Published:** 2011-12-13

**Authors:** Cecilia Perez Brandan, Angel M. Padilla, Dan Xu, Rick L. Tarleton, Miguel A. Basombrio

**Affiliations:** 1 Instituto de Patologia Experimental - CONICET, Universidad Nacional de Salta, Salta, Argentina; 2 Center for Tropical and Emerging Global Diseases, University of Georgia, Athens, Georgia, United States of America; New York University School of Medicine, United States of America

## Abstract

**Background:**

*Trypanosoma cruzi* is a protozoan parasite that causes severe disease in millions of habitants of developing countries. Currently there is no vaccine to prevent this disease and the available drugs have the consequences of side effects. Live vaccines are likely to be more effective in inducing protection than recombinant proteins or DNA vaccines; however, safety problems associated to their use have been pointed out. In recent years, increasing knowledge on the molecular genetics of Trypanosomes has allowed the identification and elimination of genes that may be necessary for parasite infectivity and survival. In this sense, targeted deletion or disruption of specific genes in the parasite genome may protect against such reversion to virulent genotypes.

**Methods and Findings:**

By targeted gene disruption we generated monoallelic mutant parasites for the *dhfr-ts* gene in a *T. cruzi* strain that has been shown to be naturally attenuated. In comparison to *T. cruzi* wild type epimastigotes, impairment in growth of *dhfr-ts^+/−^* mutant parasites was observed and mutant clones displayed decreased virulence in mice. Also, a lower number of *T. cruzi*-specific CD8^+^ T cells, in comparison to those induced by wild type parasites, was detected in mice infected with mutant parasites. However, no remarkable differences in the protective effect of TCC wild type versus TCC mutant parasites were observed. Mice challenged with virulent parasites a year after the original infection with the mutant parasites still displayed a significant control over the secondary infection.

**Conclusion:**

This study indicates that it is possible to generate genetically attenuated *T. cruzi* parasites able to confer protection against further *T. cruzi* infections.

## Author Summary

Chagas disease is the clinical manifestation of the infection produced by the flagellate parasite *Trypanosoma cruzi* and currently there is no vaccine to prevent this disease. Therefore, different approaches or alternatives are urgently needed. Vaccination with live attenuated parasites has been used effectively in mice to reduce parasitemia and histological damage. However, the use of live parasites as inmunogens is controversial due to the risk of reversion to a virulent phenotype. In this work we genetically manipulated a naturally attenuated strain of *T. cruzi* in order to produce parasites with impaired replication and infectivity, using the mutation as a safety device against reversion to virulence. We show that genetically modified parasites display a lower proliferation rate *in vitro* and induced almost undetectable levels of *T. cruzi* specific CD8^+^ T cells when injected in mice. Furthermore, the immune response induced by these live mutant parasites confers protection against a subsequent virulent infection even a year after the original immunization.

## Introduction

Chagas disease is one of the major health problems in Latin and Central America, where an estimated of 7.7 million people are infected [1]. This disease is the consequence of the infection by the protozoan parasite *Trypanosoma cruzi*. This flagellate is transmitted to mammalian hosts, including humans, by the feces of infected triatomine insects. Infection is also possible via mother to fetus during pregnancy and by contaminated blood transfusion. So far there is no effective vaccine against Chagas disease and the current available drugs have considerable side effects.

Animals surviving infection by *T. cruzi* become resistant to subsequent homologous infections. This resistance exceeds, both in strength and duration, the protection achieved with various experimental *T. cruzi* vaccines. Several naturally attenuated strains have been used in immunization-infection assays in experimental models [2], [3]. TCC is a naturally attenuated strain of *T. cruzi* that was thought to be unable to persistently infect immunocompetent mice [4]; however, recent experiments demonstrated that this strain does persist in experimental animals (Padilla AM, unpublished data). The results of immunization with this attenuated strain were promising since inoculation of live TCC epimastigotes provided protection against infection with the virulent Tulahuen strain and against each of 17 wild isolates obtained from an endemic area for Chagas in Argentina [5]. The protective capacity of this naturally attenuated strain was also evaluated in field trials against natural vector-derived infection; the TCC strain was not naturally transmitted in either guinea pigs or dogs and these TCC inoculated animals were protected against secondary natural infections [6]–[8]. Unfortunately, the potential of reversion of the TCC strain to a virulent phenotype or persistence in immunocompromised hosts cannot be foretold, rendering this method not completely safe for broad application in domestic reservoirs.

Gene targeting methods have provided a better understanding of trypanosomatid genetics, allowing the introduction or removal of specific genes from the genome of these organisms. The generation of attenuated parasites unable to sustain infection and cause pathology through removal of virulence or metabolic factors is now a reasonable possibility. A range of genetically altered parasites has been used as experimental vaccines [9], [10] but according to the literature, only four *T. cruzi* knockout lines have been evaluated as experimental immunogens. In one approach, a monoallelic mutant clone for the calmodulin-ubiquitin gene was obtained from the virulent Tulahuen strain of *T. cruzi*. Mice inoculated with different doses of mutant epimastigotes and later challenged with virulent wild type Tulahuen trypomastigotes were strongly protected, as shown by a reduction in parasite burden [11]. The second approach involved a *T. cruzi* line (L16) carrying a targeted biallelic deletion of the *lyt-1* gene. Also in this case, long-term protection against a virulent challenge was observed in mice pre-inoculated with L16 parasites as shown by a reduction in parasite load in blood [12]. In the third study, a biallelic knockout of the *gp72* gene in Y *T. cruzi* strain was shown to be highly attenuated and able to induce long lasting protection against a subsequent infection by virulent *T. cruzi* [13]. Recently, *T. cruzi* parasites lacking enoyl co-A hydratase genes (*ech1* ^+/−^ *ech*2^−/−^) were used for oral route immunization assays, showing that vaccination with genetically modified *T. cruzi* parasites confers protection against a further virulent challenge [14].

In the case of other parasitic protozoa, like *Plasmodium sp* or *Leishmania sp*, the generation of genetically attenuated parasites for use as protective vaccines has been more frequently reported [10], [15]–[18]. One particular approach was the generation of *Leishmania major dhfr-ts* null mutants. In trypanosomatids *dhfr-ts* is a single copy gene which codes for the bifunctional enzyme dihydrofolate reductase-thymidylate synthase (DHFR-TS) [19], [20]. This enzyme catalyzes sequential reactions in the biosynthesis of dTMP. Therefore inhibition of this enzyme results in thymidineless death. *Leishmania major* parasites completely lacking the *dhfr-ts* gene were generated through gene targeted deletion by homologous recombination [21]. As expected, these mutant parasites were auxotrophic and their safety and protective potential as experimental vaccines were evaluated [22]. *dhfr-ts^−/−^* parasites were able to persist in mice for up to 2 months; however, they were incapable of causing disease in both susceptible and immunodeficient mouse models. A substantial resistance to challenge with virulent *L. major* parasites was detected [22]. Moreover, heterologous protection against challenges with different *Leishmania* species was also observed [23].

Here we studied the biological effect of introducing a mutation in the *dhfr-ts* gene of the naturally attenuated TCC strain of *T. cruzi* as a safety device to avoid the potential reversion to virulent variants. Moreover, the effect of the same mutation was evaluated in *dhfr-ts^+/−^* mutant clones of the virulent Tulahuen strain. We also investigated the persistence of these parasites and their capacity to induce an immune response in infected hosts and protect against a subsequent infection.

## Methods

### Ethics statement

All animal protocols adhered to the National Institutes of Health (NIH) “Guide for the care and use of laboratory animals” and were approved by the School of Health Sciences, National University of Salta and the University of Georgia Institutional Animal Care and Use Committee.

### Parasites and culture procedures

Wild type forms of the naturally attenuated TCC and the virulent Tulahuen strains of *T. cruzi* were used, as well as two mutant clones derived from the Tulahuen strain carrying a targeted mutation of one *dhfr-ts* allele [24]. Epimastigote forms were grown at 28°C in liver digested neutralized tryptose medium (LDNT), supplemented with 10% fetal bovine serum (FBS). Metacyclic trypomastigotes were either obtained from stationary phase epimastigote cultures or by adding 1% triatomine gut homogenate [25] to epimastigote cultures and harvesting the parasites after 7 to 10 days. In both cases, complement resistant forms were purified using normal non decomplemented serum, quantified in a hemocytometer and further used to inoculate experimental animals. For the challenge experiments, fluorescent CL-tdTomato [26] as well as Tulahuen and CL wild type trypomastigotes were used. These trypomastigote forms were obtained either from Vero cell monolayers cultures or from infected mice. Infected Vero cells were cultured in RPMI 1640 medium with 10% FBS in a humid atmosphere containing 5% CO_2_ at 37°C.

### Generation of *Trypanosoma cruzi* mutant parasites

To generate parasites of the TCC strain of *T. cruzi* with a disruption of the *dhfr-ts* gene, the plasmid pBSdh1f8Neo was used. This plasmid contains the coding sequence of the *dhfr-ts* gene interrupted by the coding sequence of the neomycin phosphotransferase gene and it has been previously used for the generation of single knockout parasites, by homologous recombination, of the Tulahuen strain of *T. cruzi* [24]. Transgenic parasites were generated as previously described [24]. A total of 10^7^ early-log epimastigotes were centrifuged at 1,620 g for 10 min and suspended in 100 µl Human T Cell NucleofectorTM Solution (Lonza, Cologne) at room temperature. The resuspended parasites were then mixed with 10 µg DNA in a total volume of 10 µl and electroporated using the program “U-33” in an AMAXA Nucleofector Device (Lonza). The electroporated parasites were then cultured in 25 cm2 culture flasks with 10 ml LDNT medium and 300 µg/ml of G418 were added at 24 h post-transfection. Individual clones were obtained by single cell sorting into a 96-well plate using MoFlow cell sorter (Dako-Cytomation-Denmark).

### Molecular characterization of mutant parasites

For Southern blot analysis, genomic DNA from a selected TCC clone and from TCC wild type parasites was purified using the Phenol-Chloroform method. The DNA was then digested, separated by 0.7% agarose gel electrophoresis and the gels were blotted onto nylon membranes (Hybond-N 0.45-mm-pore-size filters; Amersham Life Science) using standard methods [27]. For probes generation, a 795 bp DNA segment corresponding to Neomycin Phosphotransferase gene was amplified from plasmid pBSSK-neo1f8 [28] using primers Neo_for (5′ ATGATTGAACAAGATGGATT 3′) and Neo_rev (5′ AGAACTCGTCAAGAAGGCGA 3′) while *dhfr-ts* gene was amplified from genomic DNA of TCC wild type parasites using primers DH5_f (5′ TGTCGCTGTTTAAGATCCGC 3′) and DH6_r (5′ CCATGAAGATGGCGGTTTAG 3′). Labeling of the probes and DNA hybridization were performed according to the protocol supplied with the PCR-DIG DNA-labeling and detection kit (Roche Applied Science).

PCR analyses were carried out using as template DNA from TCC wild type as well as TCC *dhfr-ts^+/−^* parasites. The primers used for PCR analysis were specific for the upstream gene of the *dhfr-ts* gene (PG1 5′ CTTCGAGGAGCTTTGCTGTT 3′ and PG2 5′ GATCCAACCAACTGGAGGAA 3′ ) in combination with a primer specific for the neomycin phosphotransferase gene (N 5′ GATCTCCTGTCATCTCACCT 3′).

### Epimastigote growth assays

2×10^5^ epimastigotes from mutant and wild type parasites were grown in 6-wells plates containing 5 ml of LDNT medium per well. Samples were done by triplicate and the number of growing parasites was quantified daily in a hemocytometer.

### Infectivity assays in mice

In order to evaluate the infectivity of *dhfr-ts^+/−^* mutant parasites, different mouse strains were used. C57BL/6J (B6) mice were purchased from The Jackson Laboratory. IFNγ^−/−^, Balb/c, Swiss and nude (*nu/nu*) mice (1 to 2 months old) were bred and maintained in our animal facility under specific pathogen-free conditions. Animals were inoculated by intraperitoneal (i.p.) route with metacyclic or trypomastigote forms of mutant and wild type parasites as specified.

### Immunization assays

To test the immunological protection induced by mutant clones, mice were first inoculated with 5×10^5^ *dhfr-ts^+/−^* metacyclic parasites and later challenged, at different time points, with 10^4^ blood trypomastigotes of the Tulahuen wild type strain or with 2.5×10^5^ culture trypomastigotes derived forms of the fluorescent CL-tdTomato strain [26] or CL wild type.

### Parasitological determinations

Blood (10 µl) was drawn from the tail tip of mice under slight anesthesia, and the number of parasites per 100 fields (parasitemia) was recorded from fresh blood mounts under microscope (×400). For *in vivo* fluorescence detection, footpads of mice subcutaneously infected with CL-tdTomato parasites were imaged every other day using the Maestro2 In Vivo Imaging System (CRi, Woburn, MA) with the green filter set (acquisition settings: 560 to 750 in 10 nm steps; exposure time 88.18 ms and 262 binning). Collected images were unmixed and analyzed with the Maestro software v2.8.0A. Hemocultures were performed by seeding, under sterile conditions, 200 µl of heparinized blood into 2 ml of LIT medium (Liver Infusion Tryptose) supplemented with 10% FBS. The cultures were incubated at 28°C and analyzed at day 15, 30, 45, and 60. For PCR detection of *T. cruzi*, 700 µl of blood from inoculated animals was processed following strict PCR decontamination procedures. Sample storage, DNA extraction, and amplification using primers 121 and 122 were performed as previously described [29].

### Serological determinations

Total immunoglobulin G antibodies against *T. cruzi* were measured by the enzyme-linked immunosorbent assay (ELISA) using *T. cruzi* epimastigote homogenate as antigen. The antibody concentration was expressed as the optical density at a 492-nm wavelength.

### *Trypanosoma cruzi* specific CD8^+^ T cells determination

*T. cruzi*-infected mice were bled and whole blood was stained with a MHC class I tetramer containing the *T. cruzi* specific peptide TSKB20 (TSKB20/Kb-PE tetramer) as previously described [30]. Cells were stained with anti-CD8–allophycocyanin, anti-CD11b–Cy5-PE, anti-CD4–Cy5-PE and anti-B220–Cy5-PE (all from Caltag, Burlingame, CA). CD8^+^ T cells were gated in the CD4^−^ CD11b^−^ B220^−^ lymphocyte population. Flow cytometry was carried out on a FACSCalibur flow cytometer (Becton Dickinson, San Diego, CA), and data were analyzed with FlowJo software (Tree Star, Inc., Ashland, OR).

### Statistical analysis

Continuous variables, such as antibody titers and parasite concentrations in blood samples, were analyzed with the two-tailed Wilcoxon signed-rank test for time course plots and with the Mann-Whitney or Kruskal-Wallis test for single-day measurements. Values are expressed as mean ± standard errors of the mean from at least three separate experiments. Differences between two groups were considered significant at p<0.05.

## Results

### Generation of TCC *dhfr-ts* mutant parasites

**Figure 1:**
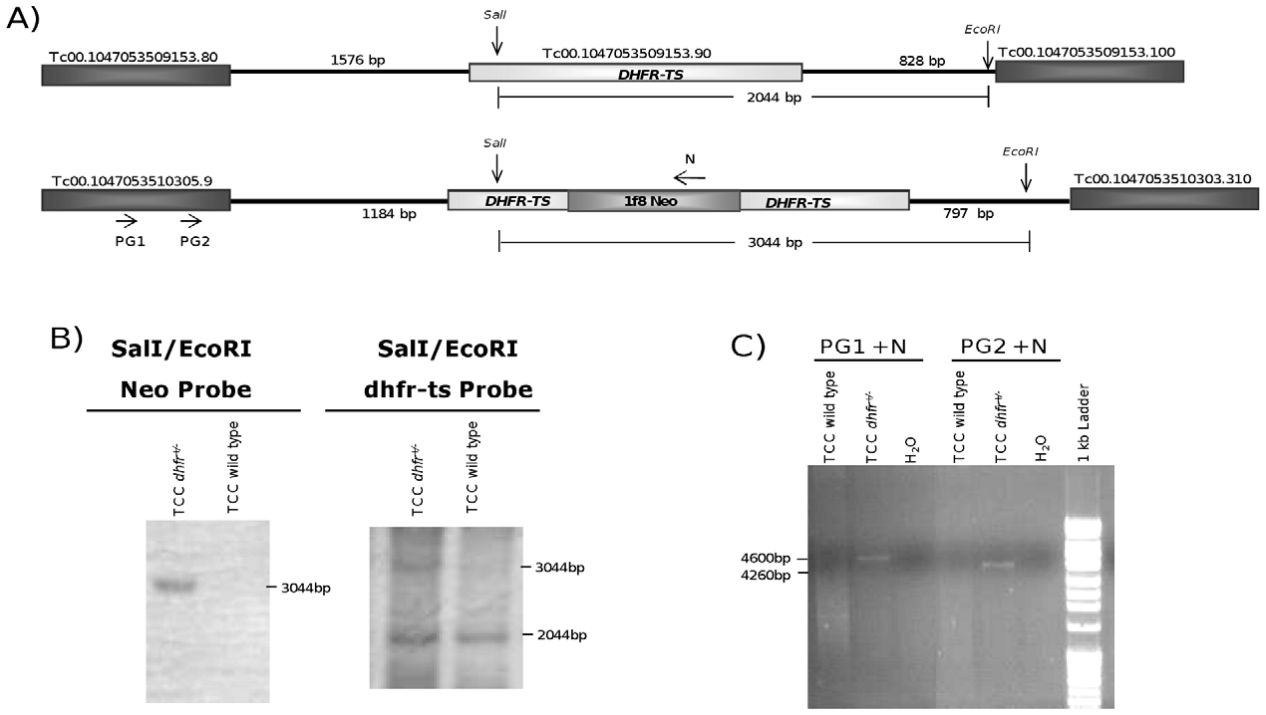
Disruption of one allele of the *dhfr-ts* gene in the TCC strain of *T. cruzi*. (A) Diagram of the expected genomic loci of *dhfr-ts* in single knockout parasites. (B) Southern Blot analysis of genomic DNA of wild type and a *dhfr-ts^+/−^* TCC clone digested by a combination of SalI/EcoRI enzymes and hybridized with a DNA probe complementary to the neomycin phosphotransferase gene or the *dhfr-ts* gene. (C) PCR analysis using a combination of specific primers complementary to the coding sequence of the upstream gene and the neomycin resistance gene.

Using constructs targeted for the interruption of the *dhfr-ts* gene, single-allele knockout parasites (*dhfr-ts^+/−^*) for the TCC strain of *T. cruzi* could easily be achieved by electroporation with the plasmid pBSdh1f8Neo and selection in 300 µg/ml of G418, as it was previously shown for the Tulahuen strain of this parasite [24]. The genome locus of *dhfr-ts* gene is shown in Figure 1A. Southern Blot analysis of a TCC *dhfr-ts^+/−^* clone confirmed the correct insertion of the neomycin phosphotransferase gene interrupting the coding sequence of *dhfr-ts* in the parasite genome (Figure 1B). By using a combination of the enzymes SalI and EcoRI, which cut outside the recombination DNA fragment electroporated and by using neomycin phosphotransferase sequence as a probe, we could confirm the correct interruption of the target gene, since a 3 kb band was obtained as expected. When hybridizing with the *dhfr-ts* probe, bands of 2 kb and 3 kb were obtained, indicating successful interruption of one *dhfr-ts* allele. PCR analyses using specifically designed primers upstream of the *dhfr-ts* gene in combination with primers for the neomycin phophotransferase gene also revealed the correct insertion of the antibiotic marker interrupting the target gene (Figure 1C). However, repeated attempts to interrupt the second copy of this gene and create null mutant parasites did not succeed, either for TCC *dhfr-ts^+/−^* or Tulahuen *dhfr-ts^+/−^* parasites. Moreover, thymidine addition to the culture media did not help in obtaining null parasites, suggesting that the *dhfr-ts* gene may be essential for *T. cruzi* survival *in vitro*. Only in one occasion and after several attempts, we were able to obtain resistance to both, neomycin and hygromycin, but these selected parasites still retained a copy of the *dhfr-ts* gene (data not shown).

### Cell growth *in vitro* is significantly affected in *dhfr-ts^+/−^* mutant epimastigotes

**Figure 2:**
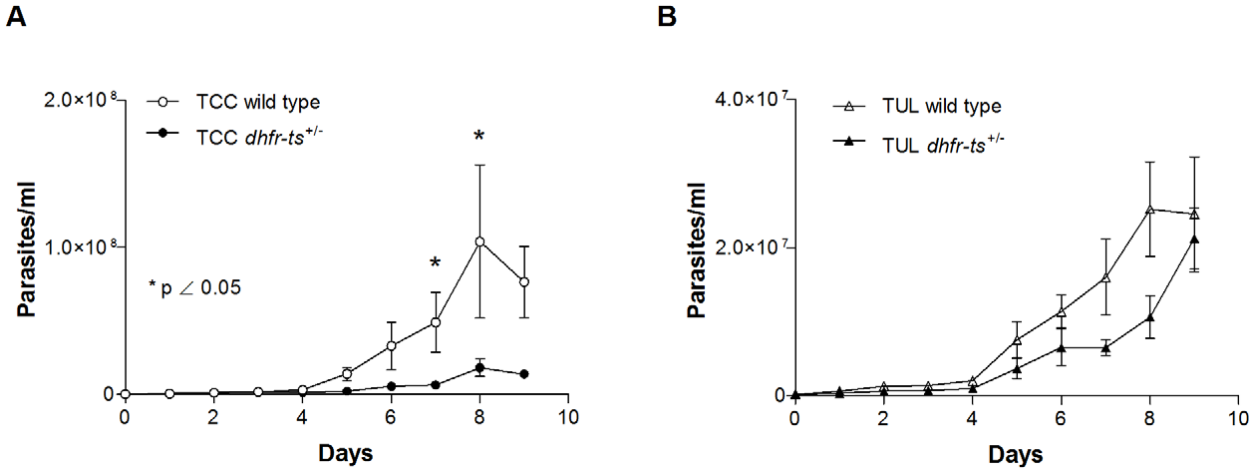
*In vitro* growth for *dhfr-ts^+/−^* and wild type epimastigotes. (A) Growth curve of TCC wild type versus TCC *dhfr-ts^+/−^* clone and (B) growth curve of Tulahuen wild type versus Tulahuen *dhfr-ts^+/−^* clone. These results are representative of 3 independent experiments.

To determine if the interruption of one allele of the *dhfr-ts* gene affects the ability of *T. cruzi* to replicate in culture, *dhfr-ts^+/−^* epimastigotes from the TCC and the Tulahuen strains, were seeded in 6-well plates in LDNT medium without selecting antibiotic pressure and parasites were counted daily until stationary phase was reached. After day 5, significant impairment in TCC *dhfr-ts^+/−^* epimastigote growth was detected when compared to TCC wild type (Figure 2A). However, these differences were less evident in Tulahuen mutant parasites when compared to Tulahuen wild type epimastigotes (Figure 2B). Addition of thymidine (100 µg/ml) did not improve mutant parasite growth (data not shown).

### *In vivo* infectivity of Tulahuen *dhfr-ts^+/−^* metacyclic trypomastigotes

**Figure 3:**
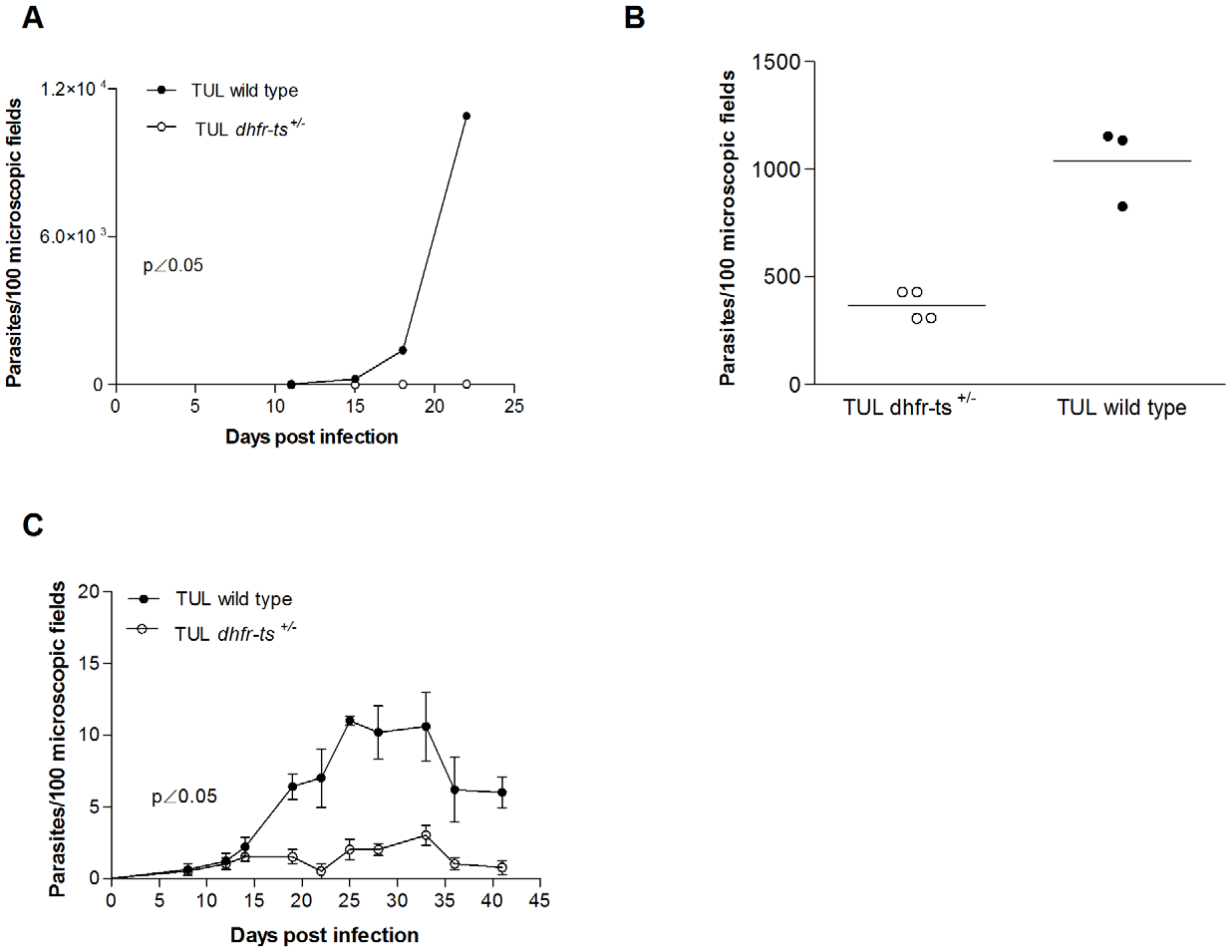
*In vivo* infectivity of Tulahuen *dhfr-ts^+/−^* and Tulahuen wild type metacyclic trypomatigotes. (A) Parasitemia curves of IFNγ^−/−^ mice inoculated with 5×10^4^ metacyclic trypomastigotes of Tulahuen wild type and *dhfr-ts^+/−^* parasites. (B) Parasite load of nude mice inoculated with 5×10^4^ metacyclic trypomastigotes of Tulahuen wild type and *dhfr-ts^+/−^* parasites at day 20 post-infection. (C) Parasitemia curves of Balb/c mice inoculated with 2×10^4^ metacyclic trypomastigotes of Tulahuen wild type and 2×10^5^ metacyclic trypomastigotes of Tulahuen *dhfr-ts^+/−^* metacyclic trypomastigotes. Values are given as means; error bars indicate standard errors of the means.

Infectivity of Tulahuen *dhfr-ts^+/−^* metacyclic trypomastigotes was determined by quantifying parasite load in blood from animals independently inoculated with either individual mutant clones or Tulahuen wild type parasites as a control. Nude mice as well as IFNγ^−/−^ mice infected with 5×10^4^ Tulahuen *dhfr-ts^+/−^* parasites succumbed after 20–25 days of infection even though the parasite load in these infected mice was significantly lower than with Tulahuen wild type parasites (Figure 3A–B). In a Balb/c mouse model differences in parasite load between mice receiving Tulahuen wild type (2×10^4^ metacyclic trypomastigotes/mouse) and mutant lines (2×10^5^ metacyclic trypomastigotes/mouse) were evident, despite the fact that 10-fold fewer wild type parasites were used to initiate these infections (p<0.05) (Figure 3C). In summary, results from three independent experiments with three different mouse strains led to the conclusion that the parasite load in mice receiving Tulahuen *dhfr-ts^+/−^* parasites was significantly lower than in mice receiving wild type parasites.

### *In vivo* infectivity of TCC *dhfr-ts^+/−^* metacyclic trypomastigotes

To determine if the naturally attenuated TCC strain could be rendered even less infective via mutation of the *dhfr-ts* gene, we evaluated the infectivity of wild type and *dhfr-ts* mutant TCC lines in different mouse strains. Since TCC parasites naturally display undetectable levels by direct blood examination in immunocompetent infected mice, the establishment of infection by TCC mutant parasites was determined by PCR and hemoculture in blood samples taken at day 15 post inoculation. No positive hemocultures were obtained from immunocompetent Balb/c or Swiss mice injected with 5×10^5^ TCC *dhfr-ts^+/−^* metacyclic parasites (Table 1). However, using nude mice infected with 10^5^ TCC *dhfr-ts^+/−^* metacyclic parasites, parasite recovery by hemoculture was demonstrated in 3/3 animals infected with TCC wild type and in 4/5 animals infected with TCC *dhfr-ts^+/−^* parasites. Lower proportions of infected animals were detected by PCR in immunocompetent Balb/c and Swiss mice inoculated with the mutant as compared to wild type TCC (Table 1). No mortality was observed in animals infected with mutant or wild type TCC parasites. Thus, the natural attenuation of TCC leaves a narrow range to measure further attenuation in the mutants. Nevertheless, in every measurable case the rates of infection obtained with TCC *dhfr-ts^+/−^* were lower than those of TCC wild type. These results led us to conclude that mutation of one allele of the *dhfr-ts* gene is sufficient to render mutant parasites less virulent than their parental line. We then wondered if these parasites were capable of surviving for long periods of time in the infected hosts; therefore we evaluated the persistence of TCC *dhfr-ts^+/−^* parasites after 60 and 120 days post infection. Day 120 samples were obtained after immunosupression with cyclophosphamide (5 doses of 250 mg/kg of cyclophosphamide per mouse and samples taken 10 days after the last dose). On day 60, all immunocompetent animals were negative by both, PCR and hemoculture, whereas 80% (4/5) nude mice still remained positive. On day 120, 3 surviving immunocompetent animals (2 Balb/c and 1 Swiss) were negative by PCR and hemoculture. Parallel determinations in TCC wild type infected animals did not differ from TCC *dhfr-ts^+/−^* in immunocompetent mice, except for the fact that in 1 out of 3 animals, a positive PCR signal was obtained. These results show that parasites are maintained below detectable levels of our most stringent techniques, opening the possibility that in some cases might even be completely clear although total parasite elimination is difficult to assess.

**Table 1:** Infectivity of TCC *dhfr-ts^+/−^* and TCC wild type parasites in different mouse strains.

Mouse strain	Hemoculture		PCR	
	TCC wild type	TCC *dhfr-ts^+/−^*	TCC wild type	TCC *dhfr-ts^+/−^*
Nude	3/3	4/5	ND*	ND*
Balb/c	0/3	0/5	2/3	2/5
Swiss	0/4	0/5	2/4	0/5

*ND: not done.

### TCC *dhfr-ts^+/−^* parasites inoculation induces a low level of specific CD8^+^ T cells

**Figure 4:**
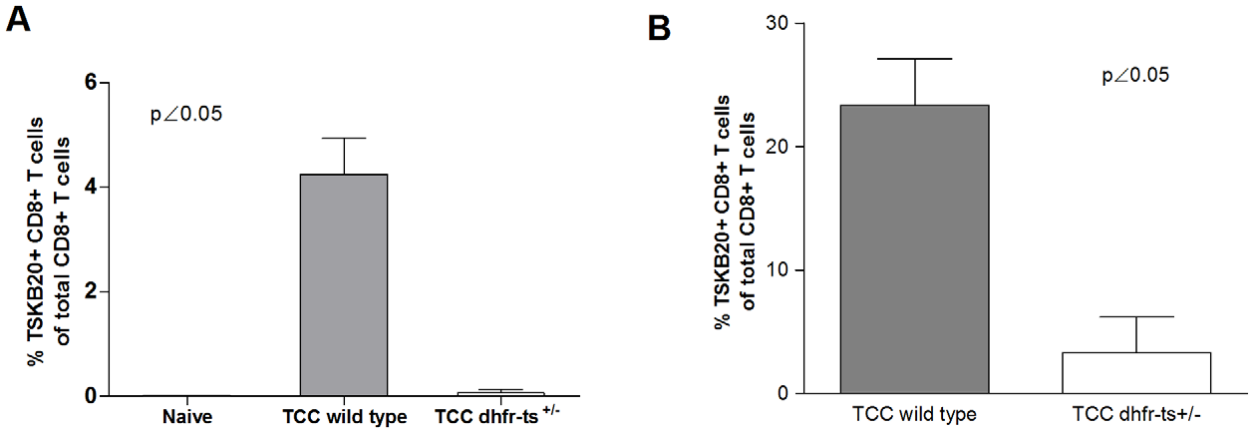
*T. cruzi* CD8^+^ specific response in mice infected with TCC *dhfr-ts^+/−^* and wild type parasites. Frequency of TSKB20-specific CD8^+^ T cells in (A) B6 mice infected with 5×10^5^ metacyclic parasites of mutant and wild type TCC parasites (n = 4) and (B) IFNγ^−/−^ mice infected with 5×10^4^ metacyclic trypomastigotes of mutant and wild type TCC parasites (n = 6 and n = 3 respectively). Bars represent the mean frequencies of CD8^+^ tetramer-positive lymphocytes per group; error bars represent standard errors of the mean.

Parasite–specific CD8^+^ T cells have been shown to be crucial in the immunity against *T. cruzi* [31]. It has been shown that the wild type TCC strain, despite being naturally attenuated, is able to induce parasite-specific CD8^+^ T cells in infected mice (Padilla AM, unpublished data). Therefore, and by staining with the MHC class I tetramer containing the *T. cruzi* specific epitope TSKB20 [30], we were able to determine the generation of specific CD8^+^ T cells in peripheral blood of mice inoculated with TCC *dhfr-ts^+/−^* and wild type parasites. For this purpose C57BL/6J (B6) mice were injected with 5×10^5^ TCC mutant parasites. Blood samples were analyzed at day 15 post inoculation. As shown in Figure 4A, CD8^+^ T cells specific response was normal in mice infected with TCC wild type parasites, while in mutant infected mice, the level of CD8^+^ T cells positive for the staining with the MHC class I tetramer containing the TSKB20 tetramer was not significantly different from naïve mice (Figure 4A). Since the attenuation of TCC *dhfr-ts^+/−^* parasites seems to be stronger than wild type parasites we analyzed the CD8^+^ response in a more sensible mouse model. For this purpose IFNγ^−/−^ mice were inoculated with 5×10^4^ metacyclic trypomastigotes of mutant and wild type TCC parasites. In this case, we also detected differences in the *T. cruzi* specific CD8^+^ T cell profile displayed 22 days after infection. The percentage of parasite-specific CD8^+^ T cell was significant lower in mice infected with TCC *dhfr-ts^+/−^* parasites when compared to TCC wild type infected ones (Figure 4B). Only one mouse infected with the *dhfr-ts^+/−^* displayed a defined MHC class I tetramer positive population different from the naive background levels and more similar to the TCC wild type infected ones. These results reinforce the previous one, demonstrating the high attenuation of the TCC *dhfr-ts^+/−^* parasites.

### Protective immunity acquired by infection with TCC wild type and *dhfr-ts^+/−^* parasites

**Figure 5:**
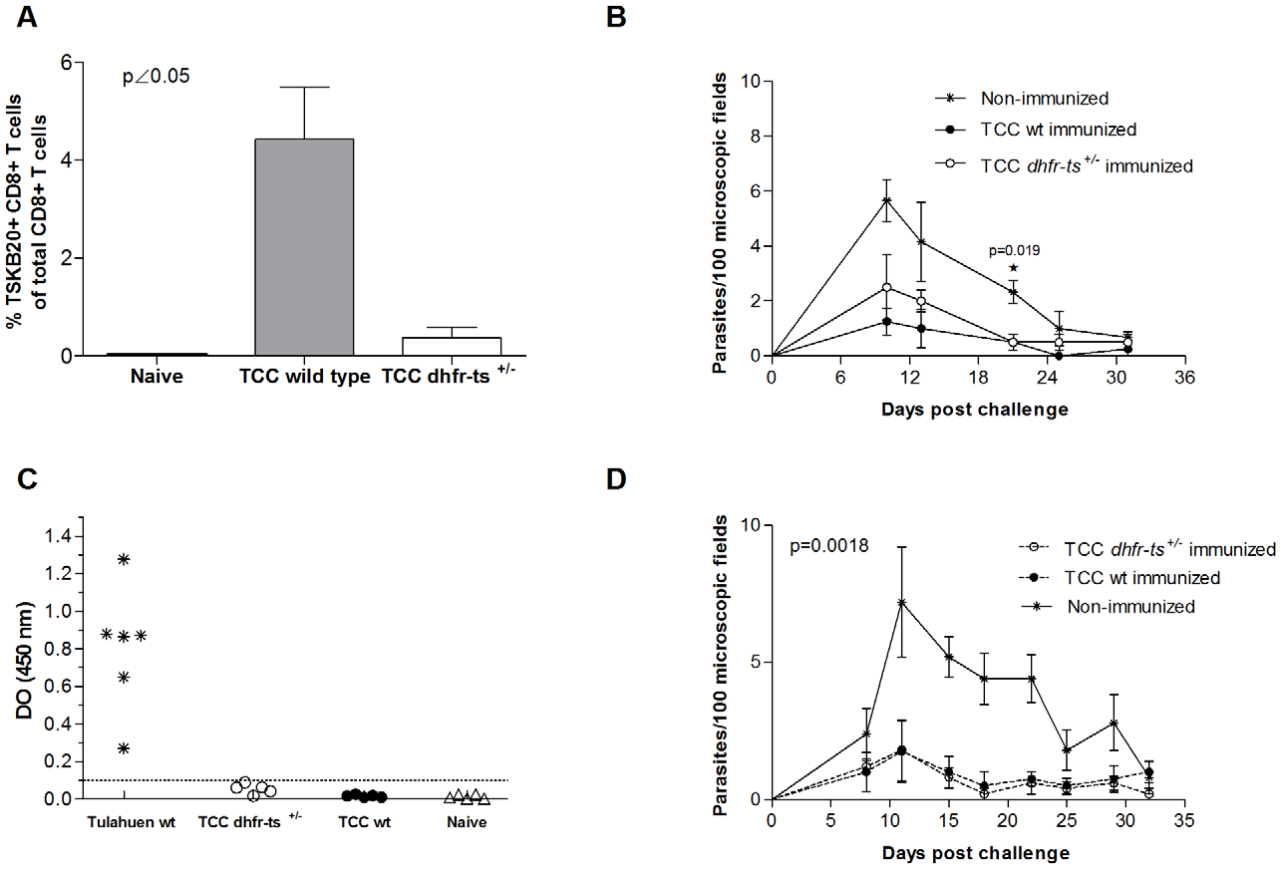
Short term protection in immunocompetent mice infected with TCC mutant parasites. (A) Lymphocytes were recovered from blood of B6 mice immunized with TCC wild type (grey bar) and TCC *dhfr-ts^+/−^* (white bar) 14 days after the boost and were stained with the TSKB20 MHC I tetramer. Bars represent the mean frequencies of CD8^+^ tetramer-positive lymphocytes for four mice per group; error bars represent standard errors of the mean. (B) Parasitemia curve of B6 mice infected with 5×10^5^ TCC *dhfr-ts^+/−^* metacyclic trypomastigotes, TCC wild type metacyclic trypomastigotes and PBS and challenge with 10^4^ virulent CL parasites. (C) Dispersion diagrams of antibody levels in either naive animals (non immunized) and those immunized with 5×10^5^ metacyclic trypomastigotes of TCC *dhfr-ts^+/−^* or TCC wild type. The results are expressed as the ratio of the absorbance of each serum sample at a 490-nm optical density (OD) to the cutoff value. Dotted lines indicate the cutoff adopted for positivity, calculated as the mean of the values determined for the naive controls plus three times the standard deviation. Positive controls were infected with Tulahuen wild type parasites. (D) Parasitemia curve of Balb/c mice infected with TCC *dhfr-ts^+/−^* metacyclic trypomastigotes, TCC wild type metacyclic trypomastigotes or PBS and challenge with 5×10^3^ virulent Tulahuen blood trypomastigotes. Values are given as means; error bars indicate standard errors of the mean.

The TCC strain of *T. cruzi* has been extensively used by our group as a live vaccine [7], [8], [32]. Since TCC *dhfr-ts^+/−^* mutant parasites displayed in several experiments a lower infectivity than TCC wild type, we tested whether this attenuation would affect the protective effect of TCC against a virulent challenge. For this purpose we carried out two independent short term immunization assays. In one experiment, groups of 4, 30-day-old C57BL/6 female mice were inoculated with either 5×10^5^ metacyclic trypomastigotes of TCC wild type, similar forms of TCC *dhfr-ts^+/−^* parasites or PBS as a control group. At day 15 post first inoculation, the animals were boosted with the same dose of parasites. To determine if this immunization regimen induced a cellular immune response, blood samples were taken during the immunization phase. In the protection assays mice immunized with the TCC *dhfr-ts^+/−^* parasites reached levels of CD8^+^ T cells specific for the TSKB20 epitope different from the naive background only after a second boost (Figure 5A). Fifteen days after the boost, the animals were challenged with 10^4^ metacyclic forms of the virulent CL strain of *T. cruzi*. Parasitemia was measured in fresh blood mounts twice a week in all animals. Mice previously inoculated with either TCC wild type or TCC *dhfr-ts^+/−^* showed a lower parasite load than challenged naïve mice (Figure 5B). Despite the lower number of specific CD8^+^ T cells detected in mice immunized with mutant parasites, no differences were found between the protection conferred by wild type and *dhfr-ts^+/−^* TCC, suggesting that the interruption of one *dhfr-ts* allele did not affect their vaccine-induced protection. Similar results were obtained in another short term immunization assay with Balb/c male mice immunized with the same doses and regimen as above but challenged with 5×10^3^ blood trypomastigotes of the virulent Tulahuen wild type strain. Specific anti-*T. cruzi* antibody levels in sera were undetectable for mice immunized with TCC wild type parasites or TCC *dhfr-ts^+/−^* (Figure 5C) and clearly different from the level for mice infected with Tulahuen wild type parasites, as determined by ELISA at 14 days post-boost. This was expected since previous results from our group showed that the TCC strain per se is not a good inducer of a humoral response [33]. However, Balb/c mice pre-infected with TCC wild type or TCC *dhfr-ts^+/−^* metacyclic trypomastigotes showed reduced numbers of circulating parasites in the peripheral blood when compared to the non immunized control group (Figure 5D). Mortality on immunized and challenged mice was null in this animal model. Again, in this experiment no differences were detected in the protective capacity of *dhfr-ts^+/−^* versus wild type TCC parasites.

### *dhfr-ts^+/−^* mutant parasites are able to confer long lasting protection against a subsequent *T. cruzi* virulent infection

**Figure 6:**
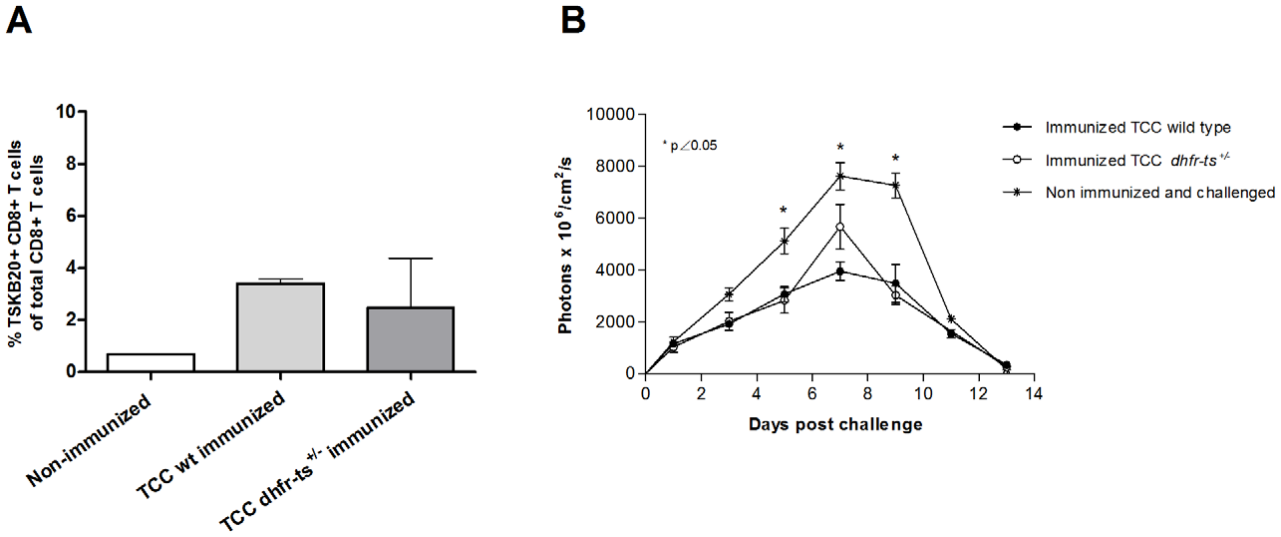
Long-term protective immunization with TCC *dhfr-ts^+/−^* metacyclic trypomastigotes against virulent challenge with *T. cruzi* CL-tdTomato. A) CD8^+^ T cells positive for TSKB20 at day 300 post infection in B6 mice inoculated with 5×10^5^ metacyclic trypomastigotes of mutant and wild type TCC parasites. B) Parasite load after challenge, at day 370 post infection, with 2.5×10^5^ bloodstream forms of the virulent CL-tdTomato strain. Fluorescence levels were measured during 13 days. Values are given as means; error bars indicate standard errors of the means.

To determine the duration of the protection observed in short term immunization-challenge experiments, we carried out a long term immunization assay. For this purpose, B6 mice immunized with TCC *dhfr-ts^+/−^* or TCC wild type parasites were challenge 370 days post infection with virulent parasites. In this case, we employed an approach of challenging with CL-tdTomato parasites expressing the fluorescent protein td-tomato [26] which can be tracked *in vivo* at the site of the infection. This technology allows us a more quantitative determination of the parasite control at the site of infection during the days following the challenge. This early determination is important since one desirable characteristic of a vaccine is to confer a rapid response and control of the parasites at the entry location, limiting their proliferation and spread through other organs. Groups of 3, 30-day-old C57BL/6 female mice were inoculated with either 5×10^5^ TCC wild type metacyclic trypomastigotes, TCC *dhfr-ts^+/−^* parasites or PBS as a control group. Blood samples were taken at day 300 post infection in order to establish the percentage of *T. cruzi* specific CD8^+^ T cells. At 300 days post infection, only one mouse inoculated with TCC *dhfr-ts^+/−^*have a detectable population of CD8^+^ T cells specific for the TSKB20 epitope. However, TCC wild type infected mice displayed a consistent TSKB20 specific population (Figure 6A). At day 370 post-infection, these mice were challenged in the footpad with 2.5×10^5^ metacyclic trypomastigotes of the fluorescent CL-tdTomato strain. Fluorescence at the site of infection was measured for 13 consecutive days as a surrogate measurement of parasite load. Figure 6B depicts the evolution of parasite load during 13 days. Mice previously infected with TCC wild type metacyclic trypomastigotes a year before were still considerably protected against the virulent challenge. Despite displaying a more attenuated behavior, TCC *dhfr-ts^+/−^* infection produced a similar protective effect compared to TCC wild type parasites. Overall, these observations indicate that both, wild type and *dhfr-ts^+/−^* TCC primo infection conferred a long-lasting protection against secondary infections.

## Discussion

Targeted gene deletion has been one of the most important tools for the study of gene functions, mainly in those organisms where the current techniques for gene silencing by RNA interference has failed [34]. The first *T. cruzi* mutant line carrying a targeted deletion of a metabolic gene was generated over 18 years ago [35]. Unfortunately, the list of genes that have been altered for reverse genetic studies in *T. cruzi* has so far not increased considerably [24], [35]–[49]. In our limited experience, the complete deletion of an identified gene through homologous recombination is not an easy task. The mutants at the *dhfr-ts* locus obtained in this work attempting to delete both copies of the *dhfr-ts* gene support this notion. Despite the correct replacement of the endogenous *dhfr-ts* gene by different antibiotic resistance genes, the presence of an extra copy in the genome may suggest an evasion strategy by the parasite to avoid the loss of this essential gene. Apparently, duplications of the target gene or the whole chromosome may be taking place. Similar events showing target locus amplification were observed when trying to obtained null mutant *T. cruzi* parasites for the enoyl-CoA hydratase (*ech*) and UDP-Glcp 4′-epimerase (TcGALE) genes [24], [38]. Identifying the frequency at which duplication events take place could be important for targeted deletion protocols and for probing the plasticity of the genome of this intriguing parasite. Possibly, trisomy and polyploidy are more frequent events than expected. Overall, our attempts to create a null mutant of the DHFR-TS enzyme strongly suggest that the *dhfr-ts* gene is essential in *T. cruzi* epimastigotes, even when exogenous thymidine is provided.

The enzyme dihydrofolate reductase thymidylate synthase of *T. cruzi* is involved in a number of different vital processes, essential for parasite survival. The impairment in *dhfr-ts^+/−^* epimastigote growth is in agreement with depletion of one allele, since the enzyme product of this gene is involved in the synthesis of thymidine monophosphate, needed for DNA assembly and therefore, for cellular replication. The significant loss of the ability of Tulahuen *dhfr-ts^+/−^* parasites to develop blood parasitism in immunocompetent mice suggests that this gene may be considered as a virulence factor of *T. cruzi*. A reduction in the virulence of knockout parasites in animal models has been previously observed in other *T. cruzi* lines. Such is the case for the *T. cruzi* Ynull line, carrying a biallelic targeted deletion of the gp72 gene. This mutation impaired the ability of Y strain parasites to maintain a latent infection in immunocompetent mice [13]. Similar results were also obtained for other *T. cruzi* mutants [11], [12], [50]. Here we report that the disruption of one copy of the *dhfr-ts* gene in the naturally attenuated TCC strain of *T. cruzi* results in even more attenuated parasites than the parental strain.

In experimental infections in mice with *T. cruzi* virulent parasites a strong CD8^+^ T cell response against immunodominant peptides encoded in trans-sialidase family genes is observed [30]. However, this specific CD8^+^ T cell response against a single epitope (TSKB20) in mice infected with TCC *dhfr-ts^+/−^* parasites was considerably lower than in mice infected with TCC wild type. The development of specific CD8^+^ T cells is determined not only by the kind but by the amount of available antigen. The lower proportion of *T. cruzi* specific CD8^+^ T cells in mice infected with TCC *dhfr-ts^+/−^* parasites could probably be correlated with the inherent propagation rate previously observed for these mutant parasites. Therefore, a late antigen presentation to dendritic cells or a lower availability of parasite antigens capable of reaching sites of priming for the CD8^+^ T cell response, may be taking place. However; both TCC wild type and TCC *dhfr-ts^+/−^* parasites, activated a protective immune response against a second virulent infection. Despite of generating a lower proportion of TSKB20 specific CD8^+^ T cells, *dhfr-ts^+/−^* parasites were able to induce protection in the immunized mice. This is in agreement with previous work showing that TSKB20 specific CD8^+^ T cells contribute to an optimal control of the acute infection, but are not crucial for the development of immune resistance [51]. Considering that the TSKB20 specific CD8^+^ T cells account for approximately 30% of the total CD8^+^ T cells in C57BL/6 infected mice at the peak of the response, it is interesting to see that TCC *dhfr-ts^+/−^* vaccinated mice are still protected, even when they display a lower proportion of TSKB20^+^CD8^+^ T cells (compared to TCC wild type infected mice) prior to challenge. This suggests that other cell populations against alternative, still undefined, epitopes may be induced by the vaccination with attenuated parasites with an important role in the protection elicited. An alternative non exclusive explanation is that the level of CD8^+^ T cell response generated and maintained by the immunization, although barely detectable may be efficient enough to crucially curb the initial replication of challenging parasites resulting in lower local and systemic parasite level. The elucidation of those mechanisms will help in defining the desired characteristics of vaccines against *T. cruzi* infection and their rational development.

A point worthy of mention is that the interruption of a copy of the *dhfr-ts* gene in the already naturally attenuated TCC strain seemed to render these parasites undetectable by highly sensitive methods after 60 days post inoculation in immunocompetent mice. Parasite recovery in low level infections is considerably difficult; thus, *dhfr-ts^+/−^* TCC parasites are not detected by a sensitive technique previously used to demonstrate parasite clearance by effective drug treatment [52] suggesting that these mutant parasites may be kept at extremely low numbers without significantly affecting their protective capacity. This result has considerable implications since if genetically modified live attenuated parasites are planned to be used in vaccination of animal reservoirs, one crucial aspect is that vaccinating parasites should be unable to be transmitted and integrated in the natural cycle. Even if mutant parasites are not completely cleared from the vaccinated animals, the considerable reduction in their number and ability to develop in the insect vector [13] should decrease the chances of being transmitted. Therefore this result opens the possibility of developing a genetically modified line with increased safety characteristics than naturally attenuated parasites without compromising the protection induced. Although targeted deletion of specific genes can be conceived as a potential approach to generate attenuated lines, genetic manipulation or complete abrogation of infectivity could lead to a loss of protective immunity. Since the immune mechanisms of protection in *T. cruzi* infection are not completely understood, it is still debatable if in the case of live attenuated vaccines, the persistence of the vaccinating parasites is required for maintaining the protection in a long term. Our results support the hypothesis that a highly controlled acute infection with genetically attenuated parasites is enough to induce a protective response which can be maintained for a long term under conditions of vaccinating-parasite persistence below detection levels or even complete clearance.
